# Effect of Metformin on Locomotor Function Recovery in Rat Spinal Cord Injury Model: A Meta-analysis

**DOI:** 10.1155/2021/1948003

**Published:** 2021-12-13

**Authors:** Qing Chen, Dong Xie, Qiuju Yao, Lili Yang

**Affiliations:** ^1^Spine Center, Department of Orthopaedics, Shanghai Changzheng Hospital, Second Affiliated Hospital of Naval Medical University, Shanghai, China; ^2^Department of Orthopaedics, No. 905 Hospital of PLA Navy, Shanghai, China; ^3^Respiratory Department, No. 905 Hospital of PLA Navy, Shanghai, China

## Abstract

**Background:**

Disorder of locomotor function is universal in patients with spinal cord injury (SCI) and has a severe impairment on their quality of life. Metformin, the first-line antidiabetic drug, has been used to improve locomotor function in SCI rats through antioxidative mechanisms recently.

**Methods:**

A search strategy was conducted from databases, including PubMed, Web of Science, MEDLINE, and Scopus database until April 2021. The methodological quality of the animal experimental studies was assessed according to the Systematic Review Centre for Laboratory animal Experimentation's Risk of Bias tool. The weighted mean difference was calculated with the random-effects model.

**Results:**

Seven eligible studies on SCI and metformin were reviewed. The meta-analysis indicated that SCI rats receiving metformin therapy showed a significant locomotor function recovery. Limitations and no obvious publication bias were presented in the studies.

**Conclusion:**

Metformin can promote the recovery of the locomotor function of SCI rats. However, the use of this meta-analysis was influenced due to the not high quality of studies. Consequently, more high-quality studies are necessary for preclinical studies of SCI in the future.

## 1. Introduction

Spinal cord injury (SCI) is a serious traumatic disease, usually caused by external mechanical injury, leading to paralysis and other serious consequences [1, 2]. The quality of life of patients with SCI is low, and SCI has caused a huge burden on families and society [1, 3]. According to reports, the incidence rate of SCI in the world is around 750 per 1 million people [4, 5]. It is necessary to find the best way to treat SCI. Surgery is often used to treat more than 70% of SCI patients, whereas usually accompanied by a poor prognosis [4, 6].

Therefore, drug therapy and cell transplantation are widely concerned and studied [7–9]. However, the pathological situation of SCI is complex, and the effect of existing drug steroid hormones is limited due to its side effects [10]. At present, the drugs studied include resveratrol, docosahexaenoic acid, and vitamins C and E, which have certain effects on the treatment of SCI, but there are still limitations [4, 7, 11, 12].

Metformin is a widely used drug to treat diabetes since the 1960s [13]. But accumulating shreds of evidence indicate that metformin could play an important role in the recovery of SCI by affecting the antioxidative mechanisms, mammalian target of rapamycin and P70S6 kinase signaling pathway, and NF-*κ*B signaling pathway [14–16]. Consequently, the efficacy of metformin for the SCI treatment is required to be studied.

We aimed to verify the hypothesis: metformin has a better curative effect than placebo on locomotor function recovery in the rat SCI model. Therefore, meta-analysis was performed to evaluate the recovery of locomotor function in SCI rats according to the Preferred Reporting Items for Systematic Reviews and Meta-Analyses (PRISMA) statement.

## 2. Materials and Methods

### 2.1. Literature Retrieval

**Figure 1:**
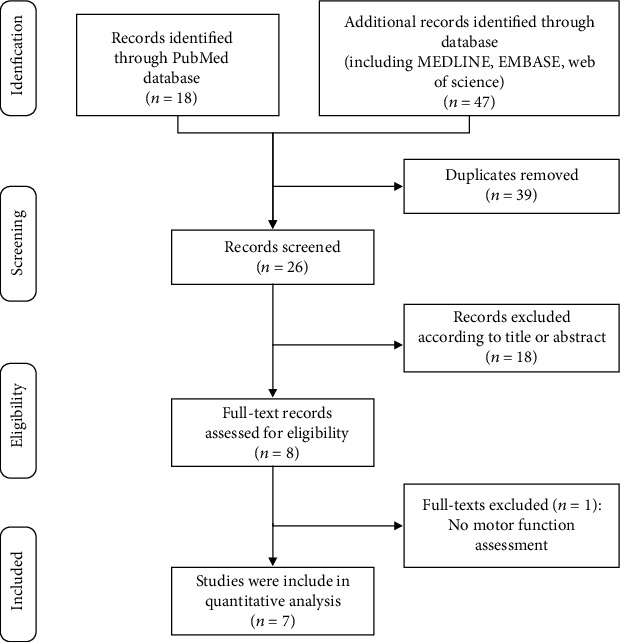
Flow diagram of the study search process in accordance with the PRISMA statement.

A search strategy was conducted from databases, including PubMed, Web of Science, MEDLINE, and Scopus database until April 2021. Relevant studies were searched using the terms “dimethylbiguanidine”, “dimethylguanylguanidine”, “glucophage”, “metformin hydrochloride”, “hydrochloride, metformin”, “metformin HCl”, “HCl, metformin”, “spinal cord trauma”, “spinal cord traumas”, “spinal cord injury”, “spinal cord injuries”, “spinal cord contusion”, “spinal cord laceration”, “post-traumatic myelopathy”, “traumatic myelopathy”, “spinal cord transection”, “rat”, and “rats”. In addition, the reference list of the relevant papers was searched for additional relevant studies. The identified studies were verified following the selected criteria by two experienced investigators individually (Figure 1).

### 2.2. Research Selection

Articles were evaluated, respectively, for preliminary screening by two reviewers in agreement with the title, abstract, and full text. Arguments discussed by a third person were resolved. The language was restricted to English research.

Researches were included according to the following criteria: (1) SCI rat models were involved with traumatic SCI, including compression and contusion injury. (2) The motor function was evaluated deliberately. (3) Intervention of metformin was compared with placebo control in SCI rats. The dose and the administration method of metformin, duration of treatment, and follow-up time were unrestricted. (4) Physiological saline, vehicle, or no treatment were included in the control groups.

Researches were excluded according to the following criteria: (1) Nontraumatic injury, penetrating injury, and complete transverse spinal cord injury of the SCI rat models were excluded. (2) The study of clinical case reports, genetically modified rats, and metformin combined with other intervention treatments was excluded. (3) Review, duplicated, and not related articles were excluded.

### 2.3. Outcome Measure

The Basso, Beattie, and Bresnahan (BBB) scale was identified as the most important outcome indicator [17]. Since the BBB scale (BBBs) was first proposed, it has been widely used in the evaluation of the functional behavior of rats. Specifically, BBBs are to score through various items (such as stepping, joint movement, trunk position and stability, paw placement, and tail position) in a still and stable evaluation environment [17, 18]. In short, BBBs are from 0 (complete paralysis) to 21 (normal movement).

### 2.4. Data Extraction

Data were independently extracted from included literature by three authors (QC, DX, and QY). The following terms were extracted: the first author, publication year, country, rat strain, weight and gender, the model and level of the SCI, the number of rats per group, the type and timing of intervention, the duration of follow-up, and the records of BBBs (Table 1). The number of rats and mean ± standard deviation of the BBBs were extracted from the metformin group and SCI control group. The outcome of each dose of the metformin treatment group was compared with the SCI control group. The last evaluation of BBBs was used. The e-mails were sent to the authors for complete data. Plot digitizer software (Free Software Foundation, Version 1.9) was explicit to extract data from the graphs if the authors were out of touch. The literature whose data were not obtainable or presented was excluded from the meta-analysis. Any controversies were resolved by discussion of a fourth author (LY).

### 2.5. Assessment of Risk of Bias in Individual Studies

The methodological quality of the animal experimental studies was assessed according to the Systematic Review Centre for Laboratory animal Experimentation's Risk of Bias tool (SYRCLE's RoB tool) [19]. The term of SYRCLE's RoB tool includes the following: selection bias, performance bias, detection bias, reporting bias, attrition bias, reporting bias, and other biases. The quality of methodology of the included studies was evaluated by two authors individually (Table 2). A “yes” indicated that the risk of bias was low, a “no” indicated the bias risk was high, and an “unclear” indicated that there were insufficient details to assess the risk of bias in the study [19].

### 2.6. Statistical Analysis

Statistics/Data Analysis 14.0 (College Station, Texas 77845, USA) was used to analyze the data extracted from the studies for meta-analysis. The weighted mean difference (WMD) was used to describe the data of the same unit, and the standardized mean difference (SMD) was used to describe the data of the different units. The 95% confidence interval (CI) was used for two types of results. *P* < 0.05 was regarded as statistically significant. The heterogeneity of included papers was evaluated by *I*^2^. The analysis of sensitivity and subgroup was used to find the source of heterogeneity. The publication bias was estimated by Egger's test and funnel plot.

## 3. Results

### 3.1. Description of Search Studies

A total of 65 studies were retrieved in the database. Thirty-nine duplicate studies were removed. Subsequently, 18 studies were excluded due to the abstract and title. Then, the full text of 8 studies was evaluated, and 1 study was excluded due to the lack of motor function evaluation [20]. Finally, 7 eligible studies were included in the quantitative analysis [14–16, 21–24]. The flow diagram of the searching process is shown in Figure 1.

### 3.2. Characteristics of Included Studies in the Meta-analysis

Characteristics of the studies included in this meta-analysis are presented in Table 1. Six studies are from China [14–16, 20–23] and 1 from Iran [24]. Five studies used female Sprague-Dawley rats [14–16, 20–22], and 2 studies used male Sprague-Dawley rats [23, 24]. All rats weighed 180-260 g. Three studies used a rat model of spinal cord compression [16, 21, 22]. Four studies reported a spinal cord contusion rat model [14, 15, 20, 23, 24]. Metformin was administered intraperitoneally in all studies. Four studies [16, 21–23] used metformin at a dose of 50 mg/kg; 1 study [14] used metformin at a dose of 10 mg/kg; 1 study [24] used metformin at a dose of 10 mg/kg, 50 mg/kg, and 100 mg/kg; and 1 study [15] used metformin at a dose of 100 mg/kg and 200 mg/kg after 2 weeks of pretreatment. All the control groups were injected with vehicles. All studies were administered immediately after SCI, and the duration of administration ranged from 1 to 28 days. BBBs were used to evaluate the locomotor function of SCI rats. The evaluation time of BBBs in 2 studies [16, 22] was 14 days, and the evaluation time of 5 studies [14, 15, 20, 21, 23, 24] reached 28 days.

### 3.3. Quality Evaluation

The SYRCLE's RoB tool was used to evaluate the bias risk in all 7 independent studies (Table 2). In general, the studies' quality was not very high. All studies described the baseline characteristics, selective outcome reporting, free of the unit of analysis errors, and design-specific risks of bias absent. One study described sequence generation [23]. Blinding was described in 2 studies [15, 24]. The free of contamination assessment was described in 2 studies [21, 22]. Only 1 study described the new animals added to replace drop-outs [15].

### 3.4. Meta-analysis of Locomotor Function Recovery of Metformin

**Figure 2:**
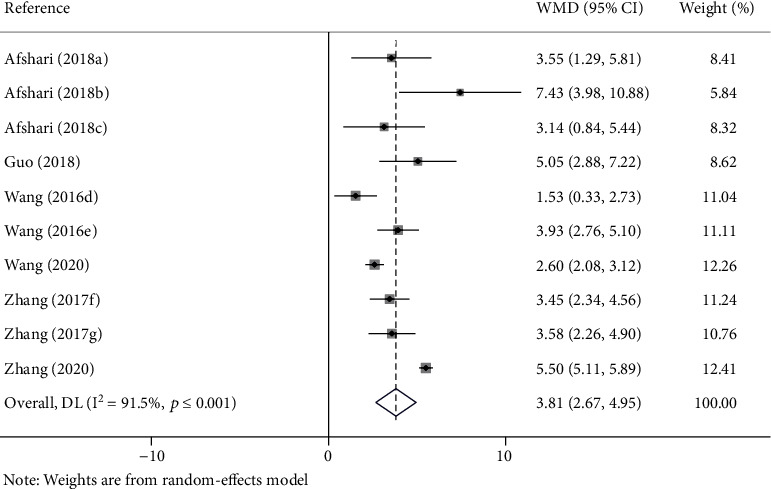
Forest plot of the effect of metformin on recovery of BBBs score in rats with SCI (random-effects models). WMD: weighted mean difference; CI: confidence interval; BBBs: Basso, Beattie, and Bresnahan scale; (a–c) the dose of 100 mg/kg, 10 mg/kg, and 50 mg/kg of metformin evaluated separately in one study; (d, e) the dose of 100 mg/kg and 200 mg/kg of metformin evaluated separately in one study; (f) the last evaluation time at the 14 days after SCI; (g) the last evaluation time at the 28 days after SCI.

The meta-analysis indicated that SCI rats receiving metformin therapy showed a significant locomotor function recovery in all studies. After the intervention of metformin, the BBBs score increased (WMD = 2.12; 95% CI, 1.37-2.88; *I*^2^ = 91.5%; *p* < 0.0001; Figure 2) in a random-effects model. After sensitivity analysis, the heterogeneity was still significant.

**Figure 3:**
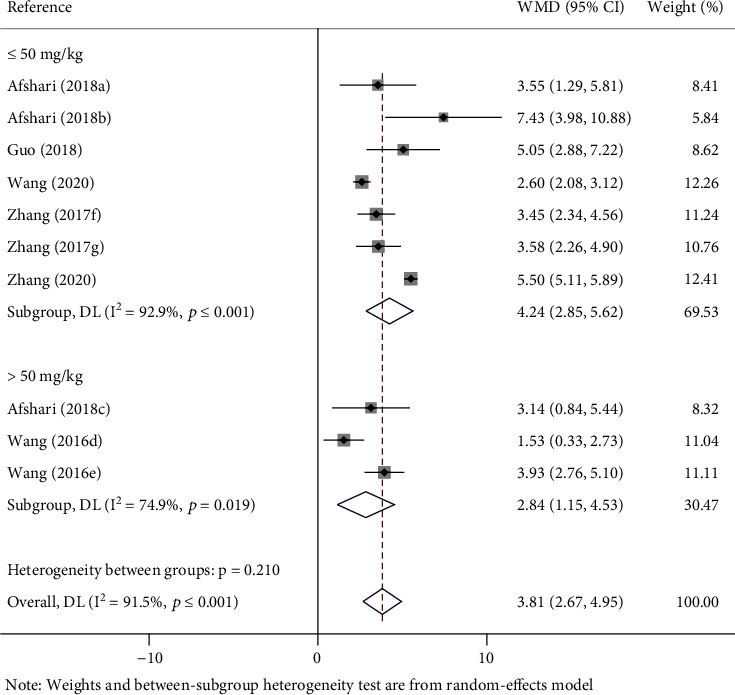
Forest plot of different doses of metformin on recovery of BBBs score in rats with SCI. WMD: weighted mean difference; CI: confidence interval; BBBs: Basso, Beattie, and Bresnahan scale; (a–c) the dose of 100 mg/kg, 10 mg/kg, and 50 mg/kg of metformin evaluated separately in one study; (d, e) the dose of 100 mg/kg and 200 mg/kg of metformin evaluated separately in one study; (f) the last evaluation time at the 14 days after SCI; (g) the last evaluation time at the 28 days after SCI.

There are different doses of metformin on recovery of the BBBs score in SCI rats. Subgroup analysis was carried out according to the metformin doses. SCI rats received metformin treatment in doses of no more than 50 mg/kg (WMD = 4.24; 95% CI, 2.85-5.62; *p* < 0.0001), and more than 50 mg/kg (WMD = 2.84; 95% CI, 1.15-4.53; *p* = 0.001) bring about a similar effect on motor function recovery (Figure 3).

**Figure 4:**
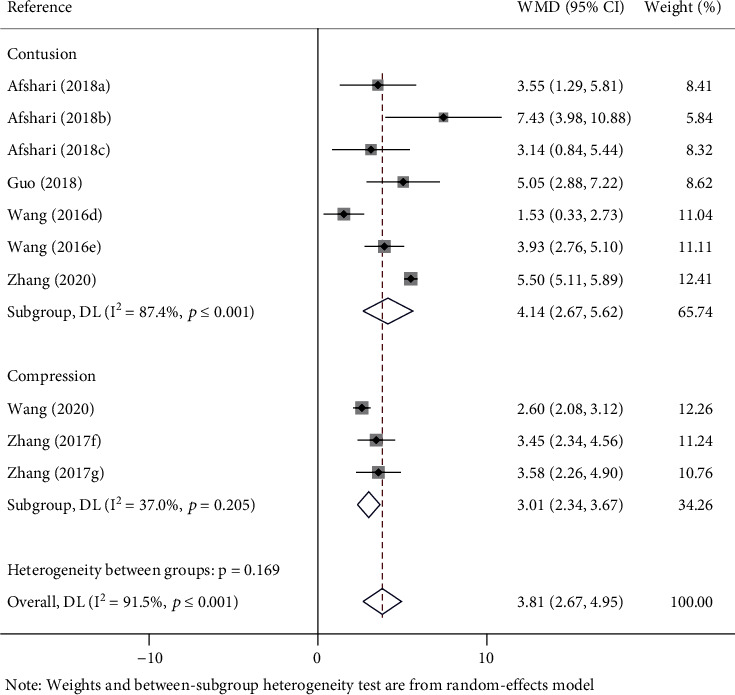
Forest plot of different models on recovery of BBBs score in rats with SCI. WMD: weighted mean difference; CI: confidence interval; BBBs: Basso, Beattie, and Bresnahan scale; (a–c) the dose of 100 mg/kg, 10 mg/kg, and 50 mg/kg of metformin evaluated separately in one study; (d, e) the dose of 100 mg/kg and 200 mg/kg of metformin evaluated separately in one study; (f) the last evaluation time at the 14 days after SCI; (g) the last evaluation time at the 28 days after SCI.

There are different SCI models of metformin on recovery of the BBBs score in SCI rats. Subgroup analysis was carried out according to different models of SCI. In 3 studies (WMD = 3.01; 95% CI, 2.34-3.67; *p* < 0.0001), BBBs of the SCI model of compression was significantly promoted in rats. In the rest of the studies (WMD = 4.14; 95% CI, 2.67-5.62; *p* < 0.001), BBBs of the SCI model of contusion was also significantly promoted in rats (Figure 4).

**Figure 5:**
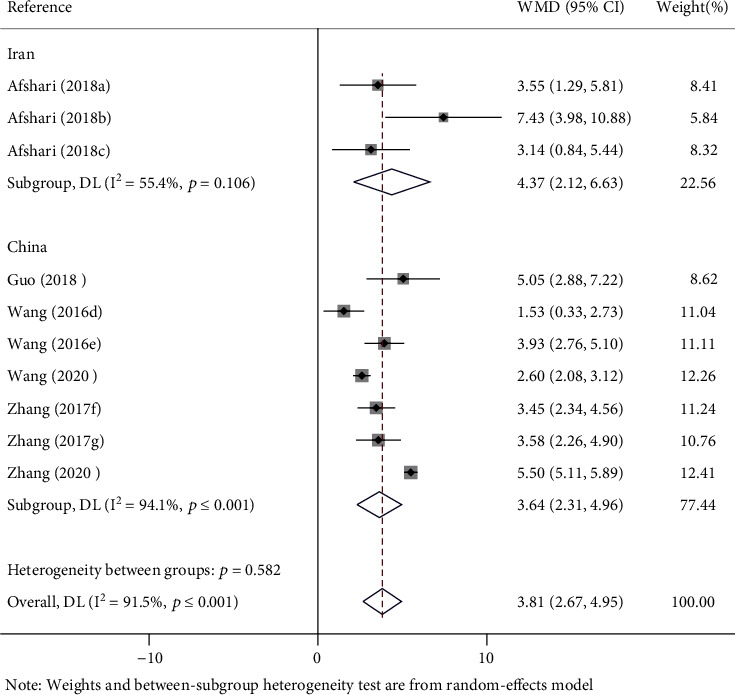
Forest plot of different countries on recovery of BBBs score in rats with SCI. WMD: weighted mean difference; CI: confidence interval; BBBs: Basso, Beattie, and Bresnahan scale; (a–c) the dose of 100 mg/kg, 10 mg/kg, and 50 mg/kg of metformin evaluated separately in one study; (d, e) the dose of 100 mg/kg and 200 mg/kg of metformin evaluated separately in one study; (f) the last evaluation time at the 14 days after SCI; (g) the last evaluation time at the 28 days after SCI.

There are different countries of metformin on recovery of the BBBs score in rats with SCI. Subgroup analysis was carried out according to different countries. One study (WMD = 4.37; 95% CI, 2.12-6.63; *p* < 0.0001) of metformin conducted in Iran on BBBs was significantly higher in rats. The rest of the studies (WMD = 3.64; 95% CI, 2.31-4.96; *p* < 0.0001) of metformin conducted in China on BBBs was also significantly promoted in rats (Figure 5).

### 3.5. Publication Bias

**Figure 6:**
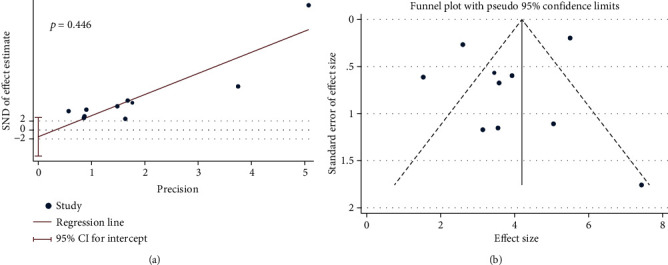
Publication bias in the studies on BBBs scores in rats after SCI: (a) Egger test; (b) funnel plot.

The Egger test and funnel plot of publication bias have been evaluated for BBBs (Figure 6). The publication bias was not shown in the Egger test and funnel plot.

## 4. Discussion

### 4.1. Outcome Profile

As far as we are aware, this is the first meta-analysis to evaluate the motor function recovery of metformin in SCI rats. Seven studies on the laboratory intervention of metformin in SCI rat models were summarized to show the preclinical effect. The meta-analysis found that the treatment of metformin of SCI rats contributed to motor function recovery. Compared with the SCI control group, a valuable promotion of the BBBs score was indicated in rats after SCI in the metformin groups. Subgroup analysis of different doses of metformin, SCI models, and countries was all performed to show the beneficial promotion of the motor function recovery after the administration of metformin. In general, the overall quality of the studies was not high, and the allocation concealment, blinding, random outcome assessment, complete outcome data, and free of inappropriate influence of funders were not reported in all studies.

### 4.2. Strengths and Limitations

This is the first meta-analysis on metformin administration in rats after SCI. Subgroup analysis of different doses, countries, and SCI models was performed to detect the effect of metformin on SCI rats. The result of animal studies is of great significance to human beings and may be used as an effective drug to restore motor function in clinical trials. All the included studies were published within 5 years.

However, some limitations exist in this meta-analysis. There is a shortage of original articles which is common in the other meta-analyses. Despite the more influential English database being searched, the relevant articles may still be omitted. The quality of the meta-analysis was not very high according to the SYRCLE's RoB tool. Publication bias, which means that the positive result and large sample are always easier to be published, erodes the authority of our study. None of the studies described allocation concealment, blinding, random outcome assessment, complete outcome data, and free of the inappropriate influence of funders. In general, the real effect is inclined to be different from our evaluated effect due to the not high quality of the involved article in this meta-analysis.

### 4.3. The Potential Mechanism for the Effect of Metformin

SCI causes secondary pathological changes, including neuronal inflammation and apoptosis, resulting in temporary or permanent spinal cord dysfunction [1, 25]. In addition, SCI also leads to lysosomal dysfunction, which leads to autophagy destruction and endoplasmic reticulum stress-induced neuronal apoptosis [26]. The neuroprotective effect of autophagy has been shown on neurodegenerative diseases in the study [27]. Some researchers found that metformin attenuates the damage of the nervous system by regulating autophagy and apoptosis after SCI [14, 16, 28]. BBBs scores significantly improved in the metformin group, compared with the SCI control group. It suggests that the administration of metformin contributes to the recovery of motor function in SCI rats through the antioxidative mechanism [16]. The potential mechanism of motor function recovery indicates that metformin would be a promising therapeutic and protective drug for SCI in terms of neurological function.

## 5. Conclusion

In general, metformin administration could contribute to the recovery of locomotor function in SCI rats according to this meta-analysis. Different doses of metformin, SCI models, and countries have similar effects. Despite the not high methodological quality, metformin would be a promising reagent of SCI treatment. A further study of metformin should be conducted in preclinical trials.

## Figures and Tables

**Table 1:** Summary of studies included in the meta-analysis.

Study	Country	Animals	Model (type and level of SCI)	Groups (*n*)	Administration time; treatment duration	Motor function; evaluation time
Wang et al. (2016)	China	Female SD rats, 180-220 g	Contusion; T9-10	A: sham (*n* = 18) B: SCI+vehicle (*n* = 18) C: SCI+met (100 mg/kg, ip.) (*n* = 18) D: SCI+MET-PC (200 mg/kg, ip.) (*n* = 18)	Group C: instantly, 3 d Group D: pretreated for 2 weeks before SCI	BBBs; 0, 1, 3, 7, 14, 21, 28 d
Zhang et al. (2017a)	China	Female SD rats, 220-250 g	Compression; T9	A: SCI+vehicle (*n* = 5) B: SCI+met (50 mg/kg, ip.) (*n* = 5)	Instantly; 14 d	BBBs; 1, 3, 7, 14 d
Zhang et al. (2017b)	China	Female SD rats, 220-250 g	Compression; T9	A: SCI+vehicle (*n* = 5) B: SCI+met (50 mg/kg, ip.) (*n* = 5)	Instantly; 28 d	BBBs; 1, 3, 7, 14, 21, 28 d
Guo et al. (2018)	China	Female SD rats, 200-240 g	Contusion; T9-10	A: sham (*n* = 20) B: SCI+vehicle (*n* = 20) C: SCI+met (10 mg/kg, ip.) (*n* = 20)	Instantly; 3 d	BBBs; 0, 1, 3, 7, 14, 21, 28 d
Afshari et al. (2018)	Iran	Male SD rats, 240-260 g	Contusion; T9	A: sham (*n* = 8) B: SCI+vehicle (*n* = 8) C: SCI+met (10 mg/kg, ip.) (*n* = 8) D: SCI+met (50 mg/kg, ip.) (*n* = 8) E: SCI+met (100 mg/kg, ip.) (*n* = 8) F: SCI+minocycline (90 mg/kg, ip.) (*n* = 8)	Instantly; 1 d	BBBs; 0, 1, 3, 7, 14, 21, 28 d
Zhang et al. (2020)	China	Male SD rats, 180-200 g	Contusion; T8-9	A: sham+vehicle (*n* = 18) B: SCI+vehicle (*n* = 18) C: SCI+met (50 mg/kg, ip.) (*n* = 18) D: SCI+met+XAV939 (0.4 mg/kg, ip.) (*n* = 18) E: SCI+methylprednisolone (30 mg/kg, ip.) (*n* = 18)	Instantly; 14 d	BBBs; 0, 1, 3, 7, 14, 21, 28 d
Wang et al. (2020)	China	Female SD rats, 220-250 g	Compression; T9	A: sham (*n* = 20) B: SCI+vehicle (*n* = 20) C: SCI+met (50 mg/kg, ip.) (*n* = 20) D: SCI+met+LY294002 (1.2 mg/kg, ip.) (*n* = 20)	Instantly; 7, 14 d	BBBs; 1, 3, 5, 7, 14 d

SD: Sprague-Dawley; met: met; SCI: spinal cord injury; ip.: intraperitoneal injection; BBBs: Basso, Beattie, and Bresnahan scale; T: thoracic vertebrae; d: day; MET-PC: metformin preconditioning.

**Table 2:** Risk of bias in included studies accessed by SYRCLE's RoB tool.

Study	Risk of bias for each item^∗^ assessed													
	Selection bias			Performance bias		Detection bias		Attrition bias	Reporting bias	Other biases				
	1	2	3	4	5	6	7	8	9	10	11	12	13	14
Afshari et al. (2018)	Unclear	Yes	Unclear	Yes	Unclear	Unclear	Yes	Unclear	Yes	Unclear	Unclear	Yes	Yes	Unclear
Guo et al. (2018)	Unclear	Yes	Unclear	Unclear	Unclear	Unclear	Unclear	Unclear	Yes	Unclear	Unclear	Yes	Yes	Unclear
Wang et al. (2016)	Unclear	Yes	Unclear	Unclear	Unclear	Unclear	Yes	Unclear	Yes	Unclear	Unclear	Yes	Yes	Yes
Wang et al. (2020)	Unclear	Yes	Unclear	Unclear	Unclear	Unclear	Unclear	Unclear	Yes	Unclear	Unclear	Yes	Yes	Unclear
Zhang et al. (2017a)	Unclear	Yes	Unclear	Unclear	Unclear	Unclear	Unclear	Unclear	Yes	Yes	Unclear	Yes	Yes	Unclear
Zhang et al. (2017b)	Unclear	Yes	Unclear	Unclear	Unclear	Unclear	Unclear	Unclear	Yes	Yes	Unclear	Yes	Yes	Unclear
Zhang et al. (2020)	Yes	Yes	Unclear	Unclear	Unclear	Unclear	Unclear	Unclear	Yes	Unclear	Unclear	Yes	Yes	Unclear

^∗^1: sequence generation; 2: baseline characteristics; 3: allocation concealment; 4: random housing; 5: blinding; 6: random outcome assessment; 7: blinding; 8: complete outcome data; 9: selective outcome reporting; 10: free of contamination; 11: free of inappropriate influence of funders; 12: free of the unit of analysis errors; 13: design-specific risks of bias absent; 14: new animals added to replace drop-outs.

## Data Availability

Datasets analyzed during the current study are available from the corresponding authors on reasonable request.
